# Injectable Chemotherapy Downstaged Oral Squamous Cell Carcinoma from Nonresectable to Resectable in a Rescue Dog: Diagnosis, Treatment, and Outcome

**DOI:** 10.1155/2018/9078537

**Published:** 2018-10-08

**Authors:** Shuang Cai, Ti Zhang, Chad Groer, Melanie Forrest, Daniel Aires, Vern Otte, Sally Barchman, Abby Faerber, Marcus Laird Forrest

**Affiliations:** ^1^HylaPharm LLC, 2029 Becker Drive, Lawrence, KS 66047, USA; ^2^University of Kansas Medical Center, 3901 Rainbow Blvd., Kansas City, KS 66160, USA; ^3^State Line Animal Hospital, Leawood, KS 66206, USA; ^4^University of Kansas, 2095 Constant Ave., Lawrence, KS 66047, USA

## Abstract

This case report documents the diagnosis, treatment, and outcome of a nonresectable oral squamous cell carcinoma in a dog with initial poor prognosis. An approximately 4-year-old female Staffordshire Bull Terrier presented with a large mass on the front of lower jaw which was diagnosed as oral papillary squamous cell carcinoma by histopathology. CT scans revealed invasion of the cancer to the frenulum of the tongue. The mass was inoperable due to location, expansiveness, and metastatic lymph nodes. The dog received 4 treatments of intralesional hyaluronan-platinum conjugates (HylaPlat™, HylaPharm LLC, Lawrence, Kansas) at 3-week intervals. Clinical chemistry and complete blood count were performed one week after each treatment and results were within normal limits. Complications included bleeding due to tumor tissue sloughing, as well as a single seizure due to unknown causes. Upon completion of chemotherapy, CT showed that the mass had regressed and was no longer invading the lingual frenulum, and multiple lymph nodes were free of metastasis. The mass thus became resectable and the dog successfully underwent rostral bilateral mandibulectomy. Over one year after chemotherapy and surgery, the cancer remains in complete remission.

## 1. Introduction

HylaPlat (HylaPharm LLC, Lawrence, Kansas) is an investigational platinum-based chemotherapeutic using hyaluronic acid as a carrier and delivery agent. In our previous studies, HylaPlat has been safely administered intralesionally to dogs and other preclinical animals without any dose-limiting nephrotoxicity, the primary side effect of intravenous cisplatin chemotherapy. Intralesionally administered HylaPlat does not result in extravasation as seen in intralesional cisplatin [1]. HylaPlat has improved pharmacokinetics and sustained retention compared to intravenous cisplatin in dogs [2]. It is effective against oral squamous cell carcinomas in dogs as demonstrated by our previous Phase I/II clinical study [3] as well as other canine clinical studies in the literature [4, 5]. Previously, HylaPlat was formulated as a liquid based injectable that had a short shelf life. Potential degradation byproduct of the original liquid formulation was toxic and lead to hepatic toxicity in previous study dogs. In the recent clinical study, we reformulated the medication to a lyophilized formulation containing sodium chloride and trehalose with improved safety profiles. The lyophilized formulation is free of the previously reported degradation byproduct. An ongoing clinical study has demonstrated the effectiveness of the chemotherapeutic. Herein, we would like to document the diagnosis, treatment, and outcome of one of the study dogs in this brief case report.

## 2. Case Description

**Figure 1:**
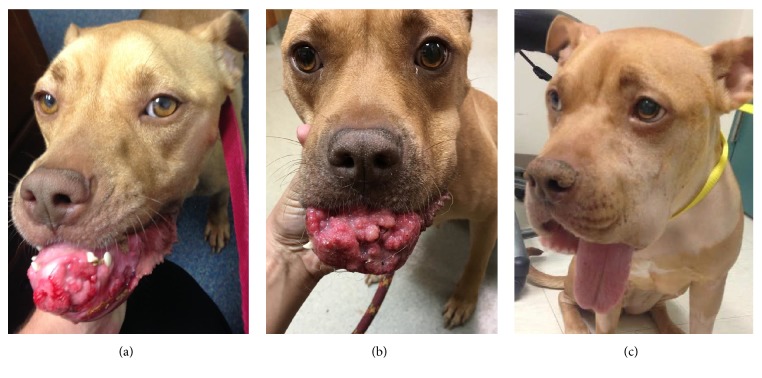
(a) A dog with an inoperable oral SCC and a metastatic lymph node before the study (lymph node staged via FNA and cytology, node not visible in image (a). (b) 3 weeks after the second HylaPlat injection. (c) After 4 HylaPlat injections, lymph node became cancer free and tumor downstaged to resectable and removed (lymph node staged via FNA and cytology, node not visible in image (c).

An approximately four-year-old female intact Staffordshire Bull Terrier was presented for evaluation of a large and fast-growing mass on the front of lower jaw, involving several teeth (Figure 1). The dog was rescued from a pound by an animal rescue shelter about 3 weeks prior to evaluation. Upon evaluation, the dog was underweight, was malnourished, and has a grade II/VI systolic heart murmur on cardiothoracic auscultation. She received a rabies vaccination and was placed on steroids, gabapentin, and antibiotics.

A large ulcerated mass was present on the rostral portion of the mandible. Full mouth dental radiographs revealed a mass that encompassed all of her lower incisors as well as her left canine and premolars. Her abdomen palpated soft and nonpainful. On palpation, her submandibular and prescapular lymph nodes were enlarged. The dog was anesthetized and a computed tomography (CT) scan of the head was performed with contrast. The CT scan revealed a large, interosseous, and expansile soft tissue attenuating mass at the most rostral aspect of the mandible, involving teeth, jaw bone, and oral membranes, and expanding almost to the frenulum of the tongue. There was a bilateral symmetrical enlargement of the mandibular lymph nodes with moderate heterogeneity following contrast. Both retropharyngeal, both prescapular and right superficial cervical lymph nodes were also enlarged. Histopathology of tissues from the mandibular mass was consistent with papillary squamous cell carcinoma. Cytology of the submandibular lymph nodes indicated reactive lymphoid hyperplasia and neoplasia. Because the mass was quite expansive and the cancer had metastasized, surgery intervention with a goal of achieving 12-15 mm margins without involving the frenulum was impossible

Shortly after diagnosis, the dog was accepted into the HylaPlat chemotherapy study sponsored by HylaPharm (Lawrence, Kansas). The dog received four intralesional injections of HylaPlat under anesthesia at three-week intervals on days 1, 22, 43, and 64. The doses ranged from 5 to 10 mg/m^2^ (mg of chemo per m^2^ of body surface area). The dog weighed 50.8 ± 1.2 lbs throughout the entire study. All four treatments went smoothly and recovery after sedation was uneventful. One hour after the first treatment, a blood sample was collected to determine the systemic exposure of the medication. Clinical chemistry and complete blood counts were performed approximately biweekly between treatments to evaluate the tolerability of HylaPlat and the results were compared to the prestudy values. Specifically, the blood samples were collected on days 0 (prior to the study), 8, 18, 26, 30, 36, 40, 47, 51, 55, 58, 61, and 68. Prior to each treatment, tumor size was measured using a caliper to monitor patient's response to the chemotherapy.

**Figure 2:**
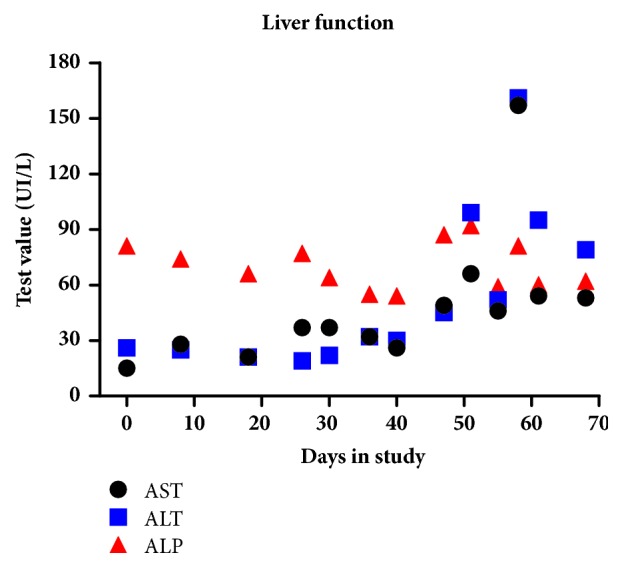
Values of liver enzymes over time. Clinical chemistry indicated that liver enzymes (AST, ALT, and ALP) were within normal limits during the entire study except on day 58 when AST and ALT were temporarily elevated and over the upper limit (AST was 157 IU/L with a REF range of 15-66 IU/L; ALT was 161 IU/L with a REF range of 12-118 IU/L). All three liver enzymes returned to normal range by day 68.

Clinical chemistry indicated that liver enzymes (AST, ALT, and ALP) were within normal limits during the entire study except on day 58 when AST and ALT were temporarily elevated and over the upper limit (AST was 157 IU/L with a REF range of 15-66 IU/L; ALT was 161 IU/L with a REF range of 12-118 IU/L). All three liver enzymes returned to normal range by day 68 (Figure 2). Total bilirubin was also monitored and the results were within the normal limits during the entire study. According to the Veterinary Cooperative Oncology Group-Common Terminology Criteria for Adverse Events (VCOG-CTCAE), a Grade I AE may be considered for the asymptomatic, transient elevation of liver enzymes as medical intervention was not indicated.

Because platinum chemotherapy may induce tubular injuries and may cause nephrotoxicity, renal function was monitored by BUN and creatinine tests during the study to assess systemic tolerability [3]. Test results demonstrated that neither BUN nor creatinine was elevated during the treatment period. Both values were within normal limits (Supplemental Figure 1). According to the VCOG-CTCAE, no renal AEs were reported.

**Figure 3:**
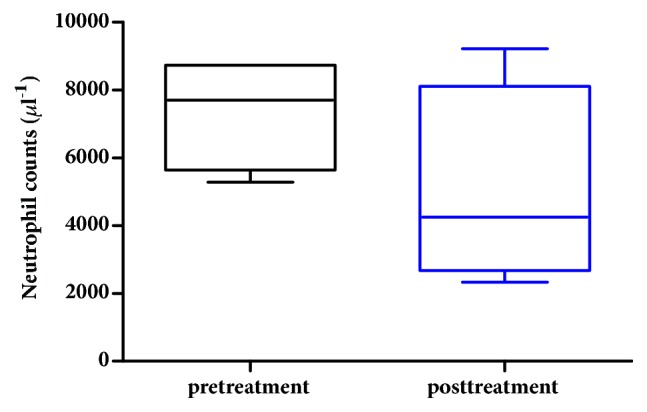
Neutrophil counts before and after chemotherapy treatments. The pretreatment result included 4 data points, each of which was generated using a sample collected before each treatment. The posttreatment result also included 4 data points, each of which was generated using a sample collected after each treatment. Result indicated that neutrophil counts at pre- and posttreatments were not statistically different (p>0.05, AVONA, GraphPad Prism).

**Figure 4:**
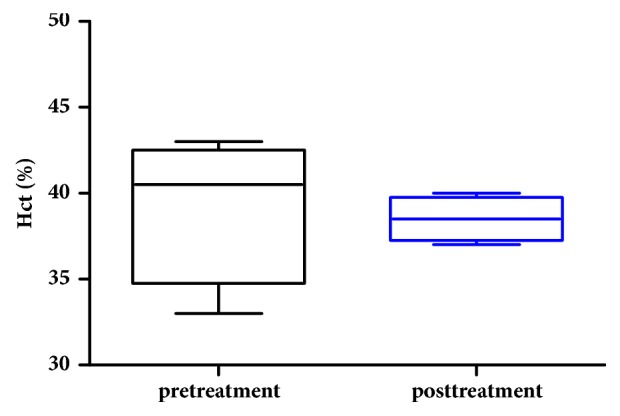
Hematocrit before and after chemotherapy treatments. The pretreatment result included 4 data points, each of which was generated using a sample collected before each treatment. The posttreatment result also included 4 data points, each of which was generated using a sample collected after each treatment. Result indicated that hematocrit did not alter significantly between pre- and posttreatments (p>0.05, AVONA, GraphPad Prism).

**Figure 5:**
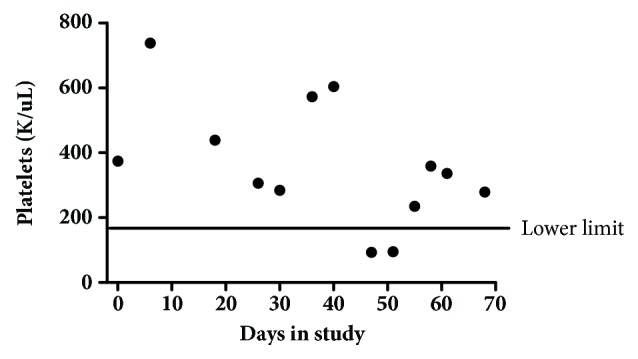
Platelet counts over time. Temporary thrombocytopenia was reported from day 4 to 8 after the third treatment. The effect was transient and the patient's low platelets resolved without medical intervention on day 12 after the third treatment.

A complete blood count with differentials was performed to determine the patient's general health status. Specifically, neutrophil and hematocrit counts are monitored to determine whether chemotherapy significantly affects hematopoietic effects and myelosuppression. Platelet count is also evaluated as myelosuppression is one of the systemic intolerabilities of platinum-based chemotherapies. Results indicated that neutrophil counts at pre- and posttreatments were not statistically different (p>0.05, AVONA, GraphPad Prism), suggesting that HylaPlat chemotherapy did not result in bone marrow suppression for this patient (Figure 3). Similarly, hematocrit did not alter significantly between pre- and posttreatments (p>0.05, AVONA, GraphPad Prism), indicating that HylaPlat chemotherapy did not significantly compromise hematopoiesis (Figure 4). Because thrombocytopenia is a potential side effect of platinum chemotherapy, the patient's platelets were monitored during the study. Temporary thrombocytopenia was reported from day 4 to 8 after the third treatment (Figure 5). The effect was transient and the patient's low platelets resolved without medical intervention on day 12 after the third treatment. According to the VCOG-CTCAE, a Grade I AE may be considered for the asymptomatic, transient thrombocytopenia as medical intervention was not indicated.

The patient received four treatments of HylaPlat at 3-week intervals. The doses were 5, 7.5, 10, and 7.5 mg/m^2^. The mass measured 5.2 cm by 5.1 cm by 3.3 cm on the day of the first treatment. After the first treatment, owner reported that the dog ate well and acted normally and no side effects were observed. Prior to the treatment the front of the tumor bled easily and after the first treatment the bleeding stopped. Three weeks after the first treatment, the dog returned for the second injection. The attending veterinarian reported that the dog's left submandibular lymph node was not as enlarged as before the first treatment on palpation. The tumor measured wider across (7.1 cm by 5.4 cm by 3.9 cm); however, the appearance of the mass improved visually with less purulent discharge and less observed bleeding. After the second treatment, the dog ate well and acted normally. Necrotic pieces of tumor tissues were observed to slough off days after the treatment. The exposed blood vessels caused by the sloughing tissues resulted in bleeding. The bleeding was managed by administration of a tranquilizer (acepromazine) to lower blood pressure and reduce activity. The dog responded to the medication and the bleeding stopped. On the day of the third treatment, the tumor measured slightly smaller on two dimensions and slightly larger on the third dimension (6.7 cm by 5.6 cm by 3.8 cm) compared to measurements made at the second treatment. After the third treatment, the owner reported that more tumor tissues fell off and the dog had heavy bleeding from the sloughing mass for 3 days. The bleeding complication may be associated with the effect of the chemotherapy (e.g., killing cancer cells, promoting tumor necrosis) as well as the friable nature of the tumor (e.g., tissue sloughing, exposing live blood vessels). Besides these symptoms, the dog acted healthy and ate well. Three weeks later, the dog received the fourth and the last injection. The tumor measured slightly smaller on two dimensions and slightly larger on the third dimension (6.5 cm by 5.5 cm by 4.8 cm) compared to measurements made at the third treatment, which we considered to be stable. Clinically, the patient was doing well; however, the tumor did bleed when it was debrided. The owner reported that the dog ate well and did well after the fourth treatment. On day 7 after the fourth treatment, heavy bleeding began from the posterior of the tumor. The dog had a single, brief seizure, the cause of which was unknown, though considerations may include blood loss, idiopathic epilepsy, liver disease, kidney failure, brain tumor, and other possible seizure-triggering conditions. The dog responded to diazepam and seizure did not recur (Grade II AE per VCOG-CTCAE). Additional diagnoses were not performed. After recovery from the ictal period, the dog was assessed as neurologically healthy.

**Figure 6:**
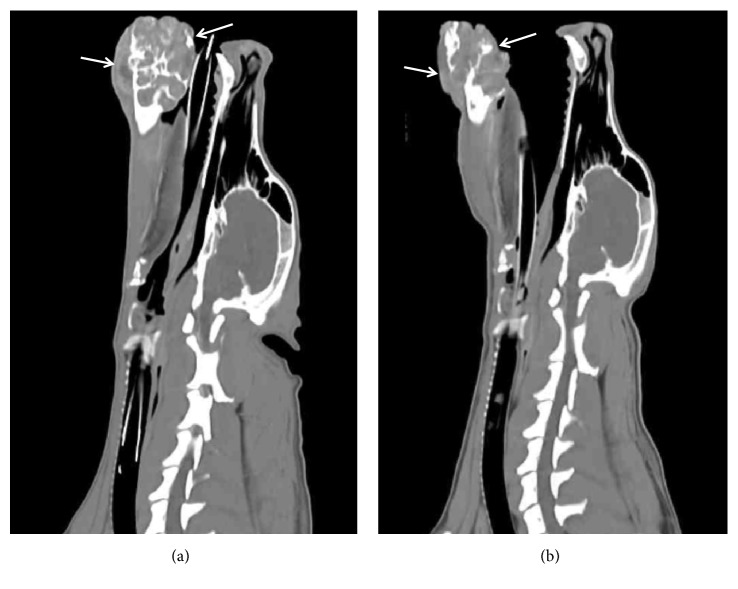
Sagittal views of computed tomography (CT) were obtained before study (a) and one month after the last treatment (b). Tumor shrinkage was observed.

Approximately one month after the last treatment, radiographs, CT, lymph node biopsy, and a physical exam were performed to determine the status of the tumor and evaluate the feasibility of a mandibulectomy to remove the SCC. Chest radiographs suggested no evidence of metastasis to the lungs. CT of the head demonstrated that the margins of the mass had regressed compared to the CT prior to the chemotherapy and were no longer invading the lingual frenulum (Figure 6). Histopathology of the enlarged mandible lymph node had no evidence of neoplasia. Based on the findings of the exams, the patient became eligible for mandibulectomy to remove the SCC.

The dog was placed under general anesthesia and underwent surgery to have an enlarged lymph node and the SCC removed. Surgery went as planned and without complication. She was laid on her left side and an incision was made on the right side of her neck to have the enlarged retropharyngeal lymph node removed. Then she was placed on her back and had most of her lower jaw removed in an effort to achieve clean margins around the SCC. The jaw was cut just in front of her first molar on the left side and just behind her third premolar on the right side. She recovered uneventfully following surgery.

Histopathology of the mandibular mass indicated that soft tissue excision was complete with clean margins. Histopathology of the right retropharyngeal lymph node was consistent with the reactive lymph node. In the days following surgery, the dog had moderate-to-severe edema and swelling around her surgical sites, which may be resulted from disruption of the lymphatic system considering the surgical locations, inflammation caused by the rostral bilateral mandibulectomy, or possible infections of the tissues and the mandibular bone at the surgical site. In an attempt to prevent infection at this site, a short course of antibiotics (Cephalexin, 500 mg capsules, one capsule every 12 hours for 7 days) was recommended. The dog was present 4 weeks after the surgery and a physical exam indicated that the healing of the surgical site was satisfactory (Figure 1). Over one year after the chemotherapy treatments and surgery, the dog is doing well and cancer remains in complete remission.

## 3. Discussion

HylaPlat (HylaPharm LLC, Lawrence, Kansas) is an injectable chemotherapy for treatment of locally advanced solid tumors, including oral SCC which is one of the most common head and neck cancers in dogs. In preclinical studies, HylaPlat has demonstrated anticancer activity against a wide range of neoplasms in mouse xenografts, including head and neck cancer [6, 7], breast cancer [1, 8], melanoma [9], and lung cancer [10]. In a Phase I pharmacokinetic and short-term tolerability study, HylaPlat has shown satisfactory tolerability and improved distribution along with sustained retention in tumor and draining lymphatics in dogs with spontaneous soft tissue sarcomas [2]. In a combined Phase I/II study in dogs with naturally occurring malignant oral and nasal SCC, 3 of 7 dogs (43%) that received low-diaqua formulations of HylaPlat demonstrated complete responses [3]. The newer lyophilized formulation in the present report appears to have improved stability and safety since the medication can be reconstituted in water just prior to administration.

According to the attending veterinarians, the lyophilized HylaPlat was readily rehydrated using sterile water in a few seconds and the intralesional injections using 23 or 25 gauge needles went smoothly without complication. To minimize environmental contamination, the medication was placed in a sealed and stoppered glass vial. Prior to use, the medication was rehydrated using Water-For-Injection by piercing the stopper. Once completely reconstituted, a proper dose was withdrawn from the vial using a syringe with a Luer Lock needle connection. The attending veterinarians and technicians were required to wear personal protective equipment (PPE) such as gloves when handling the medication.

During the course of the treatment, the patient did not experience any intolerability relating to nephrotoxicity, which is the dose-limiting toxicity of platinum-based chemotherapy. The result was consistent with previous findings from rodent, rabbit, and canine preclinical studies and canine clinical studies [1–3, 6, 8]. According to the complete blood count and clinical chemistry, the patient encountered reversible, temporary elevation of liver enzymes and depletion of platelets only after the third treatment. The changes in blood results were in good correlation with the dosages administrated as the dog received the highest dosage of 10 mg/m^2^ during the third treatment. Platinum-based chemotherapy regimens such as cisplatin and carboplatin are known to induce thrombocytopenia when used alone or in combination with other chemotherapeutic agent. Possible causes of chemoinduced thrombocytopenia may be due to the effects of the medication on stem cell proliferation, megakaryocyte progenitor cell apoptosis, platelet production, and other underlying changes in the bone marrow [11–13]. The occurrence of the changes in blood tests was not unexpected as it had occurred in an earlier canine clinical study when the previous generation of liquid HylaPlat was utilized. However, the duration and the degree of the changes in liver enzymes and platelets were significantly milder than in the previous study as a result of the improved safety of the lyophilized formulation. Despite the transient changes in blood work, the patient did not exhibit any reportable symptoms associated with possible hepatic or bone marrow toxicity. The main complication during chemotherapy was heavy bleeding from the primary lesion, which was caused by sloughing of tumor tissues and exposure of blood vessels. Overall, the chemorelated side effects were manageable.

Oral squamous cell carcinoma is the second common oral cancer in dogs. In general, tonsillar SCCs are more aggressive and metastatic than nontonsillar SCCs. Prognosis depends on the location of the tumor and the status of regional and systemic metastases. Treatment regimens depend on the location and the stage of the cancer, which include surgery with 12-15 mm margins, radiation therapy, and chemotherapy. Surgery remains the first option if the tumor is resectable and cancer has not spread to regional lymph nodes. Radiation therapy and chemotherapy are the preferred choices if cancer has metastasized or tumor is nonresectable due to either the location or the degree of invasiveness.

In this case, the patient was nonoperable as the expansive rostral mass on the mandible extended to the frenulum of the tongue, making complete surgery excision with clean margins impossible without the involvement of the tongue. Furthermore, the cancer has metastasized to the regional lymph node at the time of the diagnosis, resulting in possible poor prognosis even if partial rostral bilateral mandibulectomy was successfully performed. Thus, neoadjuvant chemotherapy was recommended with the objective of reducing both the primary mass and lymphatic penetration prior to surgical SCC removal. After four HylaPlat treatments, the primary lesion decreased in size and moved away from the base of the tongue according to the CT result, enabling surgical excision of the tumor. In addition, HylaPlat delivered the cytotoxic platinum in a sustained manner to the metastatic lymph nodes via lymphatic drainage of the primary tumor due to its engineered size (25 nm) and CD44-associated high affinity to cancer cells in the lymphatic micrometastases [14, 15]. In this case, neoadjuvant HylaPlat successfully downstaged an oral SCC patient from nonoperable to operable, facilitating life-saving excision and mandibulectomy. It may also have potentially shortened the postoperative recovery by reducing the extent of needed surgery. The impact is significant in terms of improving the prognosis and the quality of life of the patient. So far, the cancer remains in complete remission for one year at the completion of chemotherapy and surgery.

## Supplementary Materials

Supplementary Materials Supplemental Figure 1: BUN and creatinine over time. Supplemental Figure 2: the mouth and the tumor prior to the study. Supplemental Figure 3: the mouth and the tumor at 3 weeks after the first intralesional injection. Supplemental Figure 4: the mouth after the mandibulectomy. Supplemental medication list.

## References

[1] Cohen M. S., Cai S., Xie Y., Forrest M. L. (2009). A novel intralymphatic nanocarrier delivery system for cisplatin therapy in breast cancer with improved tumor efficacy and lower systemic toxicity in vivo. *The American Journal of Surgery*.

[2] Venable R. O., Worley D. R., Gustafson D. L. (2012). Effects of intratumoral administration of a hyaluronan-cisplatin nanoconjugate to five dogs with soft tissue sarcomas. *American Journal of Veterinary Research*.

[3] Cai S., Zhang T., Forrest W. C. (2016). Phase I-II clinical trial of hyaluronan-cisplatin nanoconjugate in dogs with naturally occurring malignant tumors. *American Journal of Veterinary Research*.

[4] Knapp D. W., Richardson R. C., Bonney P. L., Hahn K. (1988). Cisplatin therapy in 41 dogs with malignant tumors. *Journal of Veterinary Internal Medicine*.

[5] Shapiro W., Kitchell B. E., Fossum T. W., Couto C. G., Theilen G. (1988). Cisplatin for treatment of transitional cell and squamous cell carcinomas in dogs. *Journal of the American Veterinary Medical Association*.

[6] Cohen S. M., Rockefeller N., Mukerji R. (2013). Efficacy and toxicity of peritumoral delivery of nanoconjugated cisplatin in an *in vivo* murine model of head and neck squamous cell carcinoma. *JAMA Otolaryngology–Head & Neck Surgery*.

[7] Cai S., Xie Y., Davies N. M., Cohen M. S., Forrest M. L. (2010). Carrier-based intralymphatic cisplatin chemotherapy for the treatment of metastatic squamous cell carcinoma of the head neck. *Therapeutic Delivery*.

[8] Cohen S. M., Mukerji R., Cai S., Damjanov I., Forrest M. L., Cohen M. S. (2011). Subcutaneous delivery of nanoconjugated doxorubicin and cisplatin for locally advanced breast cancer demonstrates improved efficacy and decreased toxicity at lower doses than standard systemic combination therapy in vivo. *The American Journal of Surgery*.

[9] Yang Q., Aires D. J., Cai S. (2014). In vivo efficacy of nano hyaluronan-conjugated cisplatin for treatment of murine melanoma. *Journal of Drugs in Dermatology (JDD)*.

[10] Ishiguro S., Cai S., Uppalapati D. (2016). Intratracheal administration of hyaluronan-cisplatin conjugate nanoparticles significantly attenuates lung cancer growth in mice. *Pharmaceutical Research*.

[11] Kuter D. J. (2015). Managing thrombocytopenia associated with cancer chemotherapy. *Oncology*.

[12] Zeuner A., Signore M., Martinetti D., Bartucci M., Peschle C., De Maria R. (2007). Chemotherapy-induced thrombocytopenia derives from the selective death of megakaryocyte progenitors and can be rescued by stem cell factor. *Cancer Research*.

[13] Zhang W., Zhao L., Liu J. (2012). Cisplatin induces platelet apoptosis through the ERK signaling pathway. *Thrombosis Research*.

[14] Cai S., Xie Y., Davies N. M., Cohen M. S., Forrest M. L. (2010). Pharmacokinetics and disposition of a localized lymphatic polymeric hyaluronan conjugate of cisplatin in rodents. *Journal of Pharmaceutical Sciences*.

[15] Cai S., Alhowyan A. A. B., Yang Q., Forrest W. C. M., Shnayder Y., Forrest M. L. (2014). Cellular uptake and internalization of hyaluronan-based doxorubicin and cisplatin conjugates. *Journal of Drug Targeting*.

