# Supplementary material for: Injectable Chemotherapy Downstaged Oral Squamous Cell Carcinoma from Nonresectable to Resectable in a Rescue Dog: Diagnosis, Treatment, and Outcome
Source: Case Rep Vet Med. 2018 Oct 8;2018:9078537. doi: 10.1155/2018/9078537 (PMC6196918; doi:10.1155/2018/9078537)
Supplement: Supplementary Materials — Supplemental Figure 1: BUN and creatinine over time. Supplemental Figure 2: the mouth and the tumor prior to the study. Supplemental Figure 3: the mouth and the tumor at 3 weeks after the first intralesional injection. Supplemental Figure 4: the mouth after the mandibulectomy. Supplemental medication list. [file 9078537.f1.pdf]

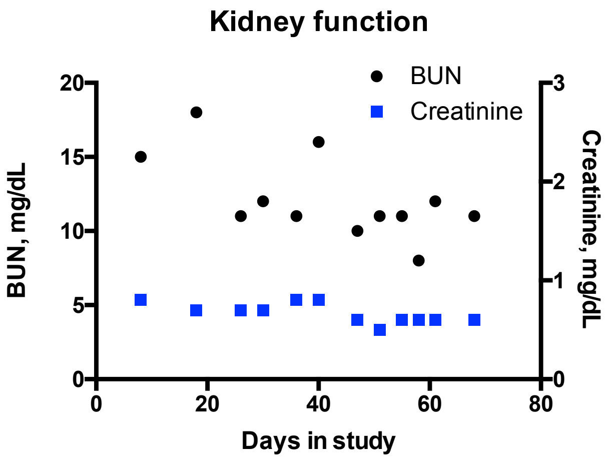

Supplemental Figure 1. BUN and creatinine over time

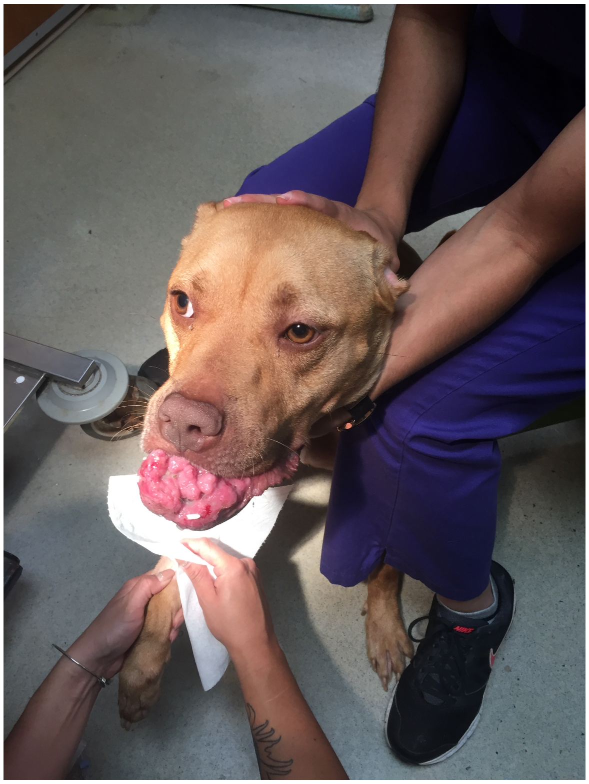

Supplemental Figure 2. The mouth and the tumor prior to the study.

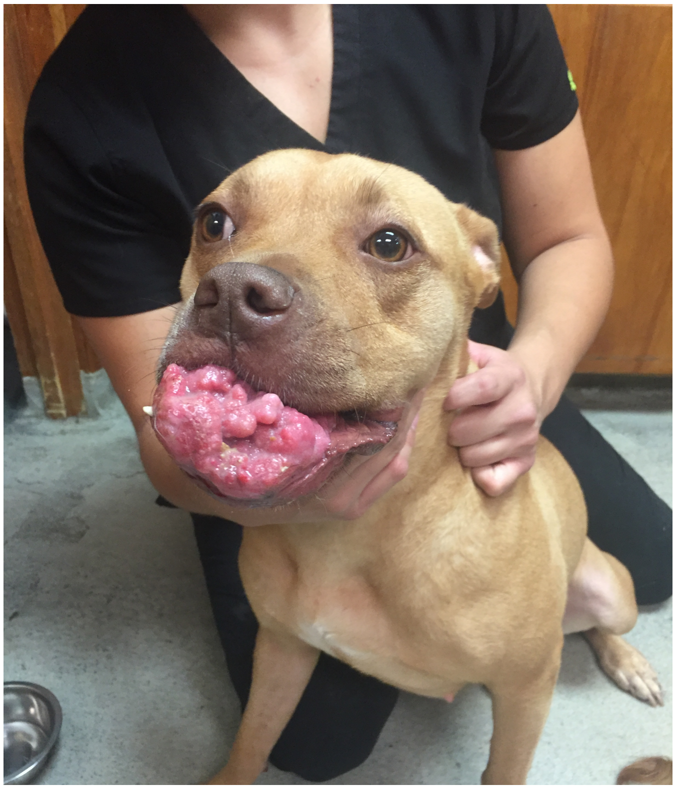

Supplemental Figure 3. The mouth and the tumor at 3 weeks after the first intralesional injection.

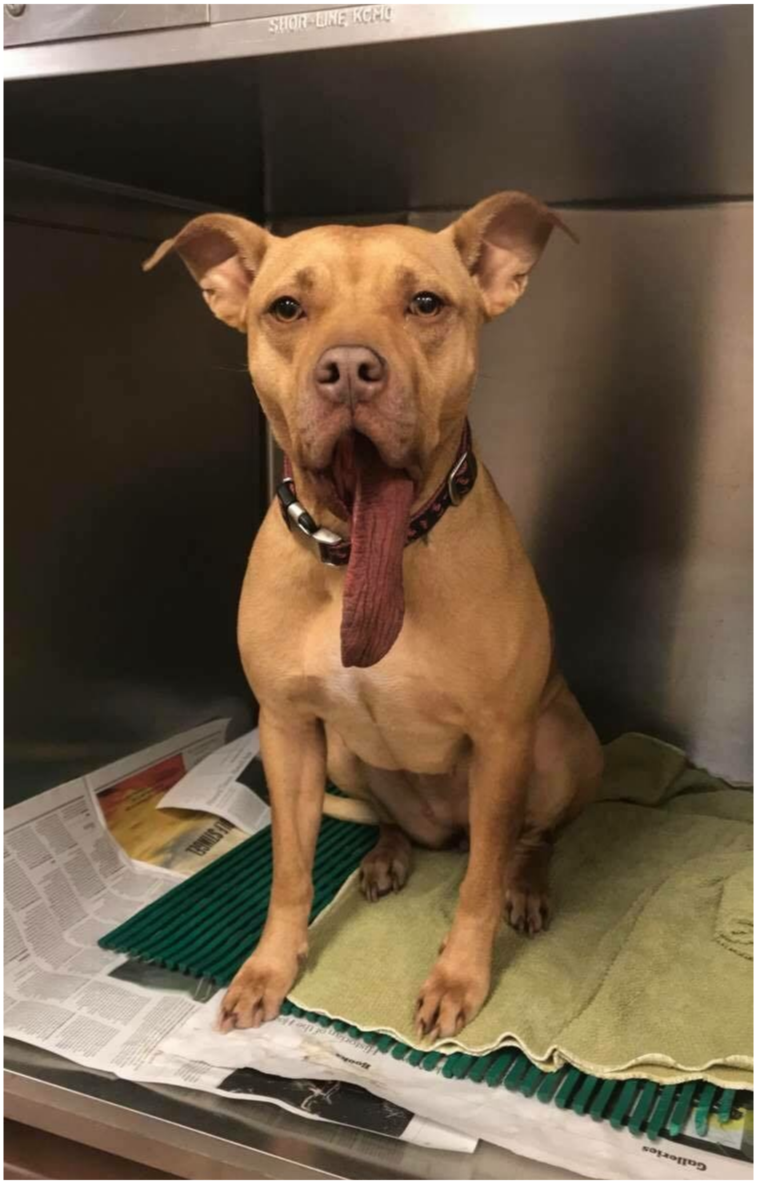

Supplemental Figure 4. The mouth after the mandibulectomy.

Medication list:

Tramadol (50 mg tablets), 2 tablets every 8-12 hours as needed for pain control.
Acetaminophen (325 mg tablets), 1.5 tablets every 8-12 hours as needed for pain control.
Carprofen (100 mg tablets), ½ tablet once every 12 hours for pain management post mandibulectomy.
Analgesic IV drip contained 2.22 mg (44.4 mL) Fentanyl in 0.9% NaCl for a total volume of 250 mL. Ran at 10 mL per hour.
Hydromorphone (2 mg/mL injection, 1 mL vial), pre-anesthetic and 3x daily for 2-3 days post mandibulectomy.
Dexmedetomidine (0.5 mg/mL, 0.23 mL) injection, pre-anesthetic
Carprofen (50 mg/mL injection), once a day for 2 days for post surgery pain management
Cephalexin (500 mg capsules), 1 capsule every 12 hours for 7 days
Clindamycin (1.5 mL injection), twice a day
Cefazolin (1 gm injection vial), 20 mg/kg once post mandibulectomy
Baytril (5 mL injection), once daily for 3 days
Propofol (10 mg/mL, 12.5 mL) for anesthetic induction before mandibulectomy
Alfaxan (10 mg/mL, 2mL) for chemotherapy administration
